# Current and future distribution of the deciduous shrub *Hydrangea macrophylla* in China estimated by MaxEnt

**DOI:** 10.1002/ece3.8288

**Published:** 2021-11-03

**Authors:** Xingyue Yan, Shuchen Wang, Yu Duan, Jing Han, Donghua Huang, Jian Zhou

**Affiliations:** ^1^ Co‐Innovation Center for Sustainable Forestry in Southern China College of Forestry Nanjing Forestry University Nanjing China; ^2^ College of Biology and Environment Nanjing Forestry University Nanjing China

**Keywords:** China, climate change, *Hydrangea macrophylla*, MaxEnt model, potential geographical distribution, shrub, variable selection

## Abstract

Climate change has a significant impact on the growth and distribution of vegetation worldwide. *Hydrangea macrophylla* is widely distributed and considered a model species for studying the distribution and responses of shrub plants under climate change. These results can inform decision‐making regarding shrub plant protection, management, and introduction of germplasm resources, and are of great importance for formulating ecological countermeasures to climate change in the future. We used the maximum entropy model to predict the change, scope expansion/reduction, centroid movement, and dominant climate factors that restrict the growth and distribution of *H*. *macrophylla* in China under current and future climate change scenarios. It was found that both precipitation and temperature affect the distribution of suitable habitat for *H*. *macrophylla*. Akaike information criterion (AICc) was used to select the feature combination (FC) and the regularization multiplier (RM). After the establishment of the optimal model (FC = QP, RM = 0.5), the complexity and over‐fitting degree of the model were low (delta AICc = 0, omission rate = 0.026, difference between training and testing area under the curve values = 0.0009), indicating that it had high accuracy in predicting the potential geographical distribution of *H*. *macrophylla* (area under the curve = 0.979). Overall, from the current period to future, the potential suitable habitat of this species in China expanded to the north. The greenhouse effect caused by an increase in CO_2_ emissions would not only increase the area of high‐suitability habitat in Central China, but also expand the area of total suitable habitat in the north. Under the maximum greenhouse gas emission scenario (RCP8.5), the migration distance of the centroid was the longest (e.g., By 2070s, the centroids of total and highly suitable areas have shifted 186.15 km and 89.84 km, respectively).

## Graphical abstract

In this manuscript, we used the maximum entropy model to predict the change, scope expansion/reduction, centroid movement, and dominant climate factors that restrict its growth and distribution in China under modern and future climate change scenarios.

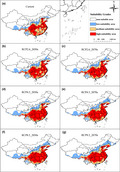

## 1 INTRODUCTION

Global climate change affects many ecosystems and biota. It not only affects the growth and distribution of global vegetation, but also leads to the loss of species diversity and germplasm resources (Kozak et al., 2008). By 2100, global temperatures are predicted to increase by 1.4 to 5.8°C from 1900, two‐ to ten‐fold higher than that in the 20th century, and the average temperature in China will rise 2.2°C by 2050 (Kumar, 2007). According to the fifth assessment report of the Intergovernmental Panel on Climate Change, global average temperature will increase 3.7°C by the end of the 21st century under the scenarios of Representative Concentration Paths (RCPs) (Lee et al., 2020). Climate variables affect vegetation composition and species distribution, and these, in turn, can directly reflect global climate change (Ni & Song, 1997). Many common species, including grasses, hybrids, and shrubs, are highly sensitive to climate change (Kane et al., 2017). The prediction of the potential geographical distribution of species based on the biological–climate relationship can not only lay the foundation for theoretical research on species origin, vegetation division, and floristic formation, but also play a significant role in genetic improvement, and species introduction and domestication (Zhang et al., 2020). It is also very important for analyzing the potential distribution of species, and for planning species protection and sustainable resource use strategies. Understanding the spatial patterns of species and their dependence on the environment is one of the basic goals in ecology and evolution (Merow & Silander, 2014). Climate models can determine the geographical distribution of biological ecosystems, and the species range is usually defined by their bioclimatic range (Christopher & Elsa, 2014). The change in species distribution range shows a polar variation in latitude and altitude to track the climate niche, which is the commonly expected result of global warming (Katherine & Monique, 2015; Laura et al., 2014), especially at high latitudes and elevations. The maximum entropy (MaxEnt) model is the most widely used tool to study species distribution and, in particular, to predict the potential future geographical distribution of species, need for only species occurrence data and environmental information (Jane et al., 2011; Phillips et al., 2006).

**FIGURE 1:**
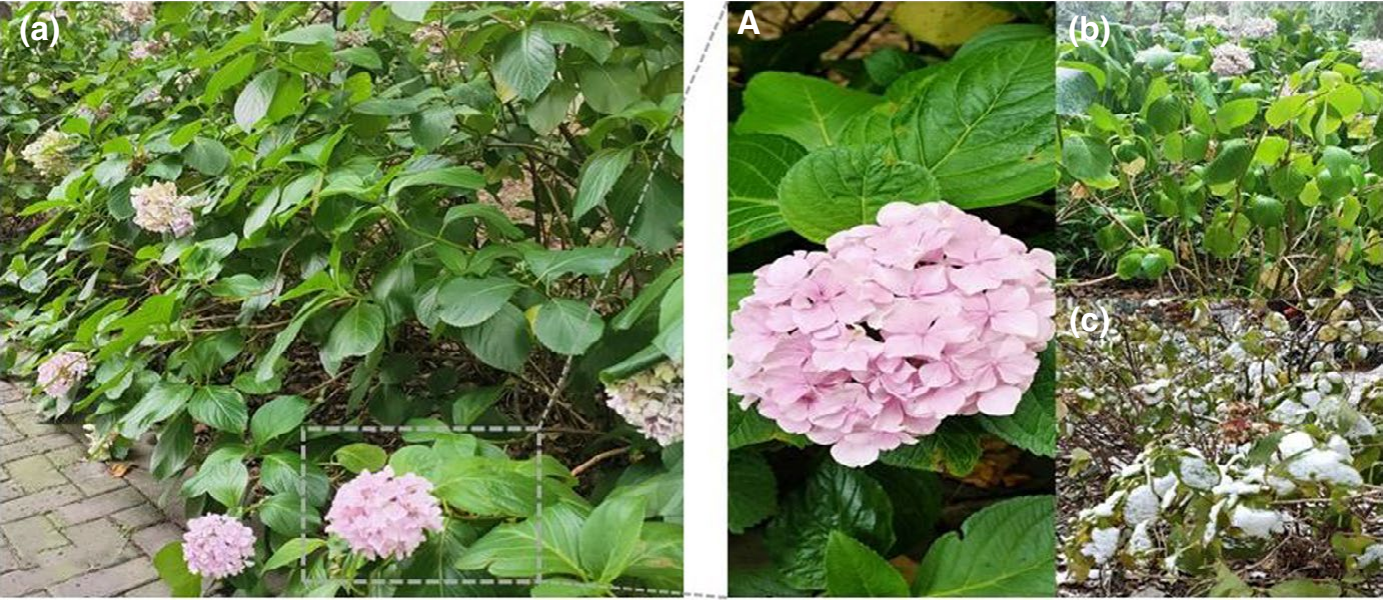
Appearance and leaves (a/A) of *Hydrangea macrophylla*. (b) Wilting leaves in summer. (c) Leaves when it snows in winter

Current research has attempted to predict the impact of future climate change on the distribution of shrub species (Bhandari et al., 2020; Xu et al., 2019). Shrubs play an important role in biogeochemical cycles, prevent soil and water erosion, provide forage for livestock, and are a source of food, wood, and non‐wood products (Hageer et al., 2017). *Hydrangea macrophylla* (Figure 1a) is a semi‐cold tolerant deciduous shrub found in the warm temperate zone (Wu et al., 2020). As a woody shrub plant, it is an important component of undergrowth in many areas and has been widely introduced globally as a horticultural plant. Presently, it is an important planting material for contemporary urban forestry construction (Galopin et al., 2010). It is named after its inflorescence that is very similar to the embroidered ball in traditional Chinese culture; its inflorescence is large, rich in color, and has high ornamental value. It is known as one of the flowers with great development potential (Zeng et al., 2018). In summer, even growing in the shady environments of Nanjing, Jiangsu Province, the leaves of plants are prone to wilting due to long‐term arid conditions (Figure 1b). In winter, the process of defoliation is not completed until the middle of December (Figure 1c). Its natural distribution areas, and introduction and cultivation areas have spread globally, including in the temperate zone, warm temperate zone, north subtropical zone, and south subtropical zone (Zeng et al., 2018). Therefore, *H*. *macrophylla* is an ideal model species for studying the distribution of plants and their response to climate change. The *Hydrangea* genus in China is rich and diverse, and the country is considered the center of *Hydrangea* germplasm resources. Most of the parent species of the cultivated species are distributed in China, and the cultivation history is very long. According to literature, the cultivation research of *H*. *macrophylla* in China could be traced back to the Tang Dynasty, and was introduced to England in the 18th century (Ceng et al., 2018; Qiao et al., 2020). Records show that there are approximately 73 species of *H*. *macrophylla* globally, with 47 species and 11 varieties in China (Cai et al., 2018). At present, there has been some research conducted on the response of *H*. *macrophylla* to high and low temperature stress and drought (Cai et al., 2018; Zhang, Li, et al., 2018; Zhang, Meng‐qi, et al., 2018), but its distribution, limiting factors, and possible response to future climate change in China have not been reported. This study was the first attempt investigating the spatial distribution of this plant species in China as a whole. These are important basic data for the development of *H*. *macrophylla* planting, scenic spot planning, and layout division, and therefore, it is important to collect such data considering the abundant germplasm resources of *H*. *macrophylla* in China.

In combination with the literature and sample collection records of *H*. *macrophylla*, the MaxEnt model was used to predict the potential distribution of this species under three different RCPs (RCP2.6, RCP4.5, and RCP8.5) under current (1960–1990) and future conditions: 2050s (2041–2060) and 2070s (2061–2080), and to explore the key environmental factors causing potential geographical distribution changes. The purpose of this study was to further understand the species distribution characteristics and ecological adaptability of small deciduous shrubs, and to provide theoretical reference for the protection and utilization of the wild resources of *H*. *macrophylla*, as well as future large‐scale introduction and cultivation (Qin et al., 2020). The main objectives of this study were to: (1) understand the geographical distribution pattern and future changes of *H*. *macrophylla* in China; (2) determine the dominant climatic factors that restrict the growth and distribution of *H*. *macrophylla*; (3) predict the suitable habitat of *H*. *macrophylla* and its centroid trajectory under current and future climate change scenarios; and (4) provide a scientific basis for the protection and introduction of *H*. *macrophylla* resources.

## 2 MATERIALS AND METHODS

### 2.1 Data

#### 2.1.1 Species records data

Through the Chinese Digital Herbarium (http://www.cvh.ac.cn/), Chinese Plant Image Database (http://ppbc.iplant.cn/special), Global Biodiversity Information Service Network Platform (https://www.gbif.org/), and other digital platforms, we extracted the specimen data of *H*. *macrophylla* from China. We used Baidu tools (http://api.map.baidu.com/lbsapi/getpoint/index.html) to confirm the geographic coordinates of each distribution record, the specimens with no locational coordinates and duplicate data were eliminated (Huang et al., 2018). To reduce the error of the clustering effect in predicting the potential distribution region and reduce model over‐fitting, we only selected one point (or sample record) in the 2.5′ × 2.5′ grid range (Xie, 2011). Finally, we selected 307 points for further analysis.

#### 2.1.2 Environmental data

Weather data were downloaded from WorldClim (http://www.worldclim.org/), all of them included 19 climatic variables (*Bio 1*–*Bio 19*), and the spatial resolution was 2.5′. The current climate data selected from 1960 to 1990 (version 1.4). The CCSM4 climate system model data released by the Coupled Model Intercomparison Project Phase 5 stage (Coban et al., 2020) are used for future climate data. The RCP scenarios consist of four pathways, including RCP2.6, RCP4.5, RCP6.0, and RCP8.5 (Huang et al., 2020, Remya et al., 2015). RCP4.5 and RCP6.0 are both moderate greenhouse gas emission scenarios, and RCP4.5 has a higher priority than RCP6.0 (Wei et al., 2018). Therefore, we chose RCP2.6 (minimum greenhouse gas emission scenario), RCP4.5 (moderate greenhouse gas emission scenario), and RCP8.5 (maximum greenhouse gas emission scenario) to simulate the current suitable habitat distribution of *H*. *macrophylla*.

### 2.2 Data analysis

#### 2.2.1 Selection of environmental variables

In order to avoid correlation between various climate variables, which leads to over‐fitting and affects the accuracy of prediction, 19 bioclimatic variables data from 307 data points were first extracted using QGIS v.3.14, and then imported into R v.3.6.3. Pearson correlation analysis of data was carried out using the *cor* function in R (Wang, Li, et al., 2018; Wang, Wu, et al., 2018). For highly correlated variables (correlation coefficient >0.8), only one of the more suitable variables for model interpretation was selected as the representative variable to participate in subsequent model prediction (Liu & Shi, 2020; Radosavljevic & Anderson, 2014). Finally, 10 biological variables were selected from 19 biological variables to participate in the model prediction (Table 1).

**TABLE 1:** Environmental predictors selected in model building after correlation analysis

Variable code	Climate variables (units)	Variable code	Climate variables (units)
Bio2	Mean diurnal range (°C)	Bio8	Mean temperature of wettest quarter (°C)
Bio3	Isothermality (×100) (–)	Bio12	Annual precipitation (mm)
Bio4	Temperature seasonality (standard deviation ×100) (–)	Bio15	Precipitation seasonality (–)
Bio6	Min temperature of coldest month (°C)	Bio17	Precipitation of driest quarter (mm)
Bio7	Temperature annual range (°C)	Bio18	Precipitation of warmest quarter (mm)

#### 2.2.2 Optimization of model parameters and model building

The predictive performance of the model is influenced by two parameters that require optimization: (i) the regularization multiplier (RM) and (ii) the feature combination (FC). Optimization was achieved using the kuenm package (Cobos et al., 2019) in R v3.6.3. In the optimization process, 307 distribution records were divided into four equal parts by block method, including three for training and one for testing. Then, we set RM constants 0.5 to 4.0 with steps of 0.5 (Li et al., 2020). For the selection of FC, we selected 29 combinations of five feature classes (linear = l, quadratic = q, product = p, threshold = t, and hinge = h) for testing, including L, LQ, LQH, and LQHPT, etc (Appendix 1). Finally, the above 232 candidate models were tested by kuenm package. Best models were selected according to the following criteria: (1) significant models with (2) omission rates ≤5% (Cobos et al., 2019). Then, the model with the minimum AICc value (i.e., delta AICc = 0) was considered as the final model, and all models with delta AICc <2 had high reliability (Li et al., 2020; Wang, Li, et al., 2018; Wang, Wu, et al., 2018).

Then, we saved 307 records in.csv format initially, and then imported the sample distribution information and the 10 bioclimatic variables obtained after screening into the MaxEnt model v.3.4.4 for distribution simulation simultaneously. In order to enhance simulation accuracy, the sample data were randomly divided into a test data set (25%) and a training data set (75%) (Hu et al., 2014). The final output result file was an average of 10 repetitions. Finally, we evaluated the main ecological factors affecting the distribution of *H*. *macrophylla* according to the contribution rate of environmental factor variables and the results of the jackknife test. The area under the curve (AUC) value of the receiver‐operating characteristic curve (ROC) is a comprehensive index reflecting the sensitivity and specificity of a model (Gebrewahid et al., 2020). In practical applications, the AUC index derived from ROC is commonly used to evaluate the model performance index (Xu et al., 2020). Since the AUC value is unaffected by the threshold value, it is a more reasonable basis for comparing different models. When the AUC value exceeds 0.8, the simulation model is accepted. For a better simulation model, this value should exceed 0.9 (Li et al., 2020; Sun et al., 2020; Zhang, Li, et al., 2018; Zhang, Meng‐qi, et al., 2018). In addition, AICc value and the difference between training and testing AUC values [AUC. Diff] comprehensively reflect the goodness of fit and complexity of the model, both of which are an excellent standard to measure the performance of the model (Cobos et al., 2019).

#### 2.2.3 Classification of suitable habitat

The data generated by MaxEnt software were imported into ArcGIS v.10.6. The conversion tool was used to convert the data into raster data, and then the classification of suitable *H*. *macrophylla* habitat was carried out using the reclassify tool (Hu & He, 2020). According to the method of artificial classification, the distribution areas of *H*. *macrophylla* were classified as follows: unsuitable area (0 ≤ *p* ≤ .1), low‐suitability area (.1 < *p* ≤ .3), medium‐suitability area (.3 < *p* ≤ .5), and high‐suitability area (.5 < *p* ≤ 1) (Qiu et al., 2020). Finally, we obtained the potential distribution of suitable habitat for *H*. *macrophylla* in China (*p* > .1).

#### 2.2.4 Changes in suitable habitat area and centroids

To further examine trends, the centroids of the current and future climate distribution areas were calculated using the Python‐based GIS toolkit, SDMtoolbox (Brown et al., 2017). We imported.asc files generated under different emission scenarios predicted by the MaxEnt model into ArcGIS v.10.6, and used the SDMtoolbox to analyze and map the spatial pattern and centroid changes of the suitable habitat area of *H*. *macrophylla*. In order to further analyze the changes under current and future climate change scenarios, we used centroid position changes to reflect the change in the direction of the total and high‐suitability areas, and evaluated the migration distance of the suitable habitat area of *H*. *macrophylla* on longitude and latitude coordinates.

## 3 RESULTS

### 3.1 Optimal model and accuracy evaluation

The Maxent model was used to simulate the potential distribution area of *H*. *macrophylla* in China. When the model was set with the default parameters, delta AICc was 126.56, but when it was set with the optimization parameters (FC = QP and RM = 0.5), delta AICc was 0 (Table 2). The AUC. Diff decreased by 67.86% and the omission rate decreased by 75%, indicating that the over‐fitting degree of the optimization parameters setting was lower than that of the default parameters. Using the optimized parameters (FC = QP and RM = 0.5) to reconstruct the model and simulate the suitable area of *H*. *macrophylla* in China, the average value of training AUC reached 0.979. The simulation results of the software indicated that the MaxEnt model was very accurate in predicting the geographical distribution of *H*. *macrophylla* in China.

**TABLE 2:** Evaluation metrics of the default and optimal Maxent models by kuenm package

Setting	FC	RM	AUC. Diff	Omission rate	Delta AICc	AICc
Default	LQPTH	1	0.0028	0.104	126.56	7597.30
Optimized	QP	0.5	0.0009	0.026	0	7505.92

### 3.2 Current potential distribution area

The distribution area of *H*. *macrophylla* in China is 88°55′–121°47′E, 18°44′–40°32′; it is mainly concentrated in southern and Central China. Its distribution covers Sichuan, Shaanxi, Yunnan, Hunan, Hubei, Jiangxi, Guizhou, Henan, Hebei, Gansu, Shandong, Fujian, Guangdong, Shandong, Jiangsu, Anhui, Zhejiang, Hainan, Taiwan, Hong Kong, Inner Mongolia, Guangxi and Tibet, Beijing, Chongqing, Shanghai, and Tianjin.

**FIGURE 2:**
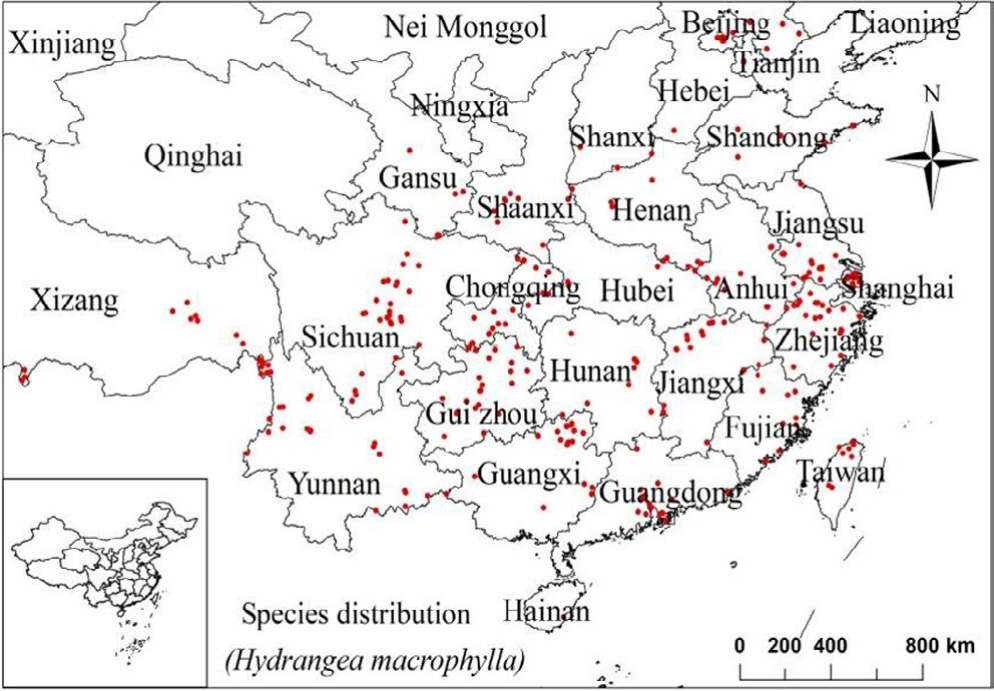
Distribution of the occurrence points of *Hydrangea macrophylla* in China

Almost all of the sample points screened were scattered in the suitable habitat area of *H*. *macrophylla* (Figure 2). This indicated that the model could simulate the potential distribution of *H*. *macrophylla*. According to the calculation results of the prediction model and the actual distribution of sampling points, it can be seen that, due to the limitation of climatic conditions, northwest and northeast China is not suitable for the growth of *H*. *macrophylla* (accounting for 63.53% of the national area, a total of ca. 6,120,677/km^2^). The regions included northern and western Tibet, Xinjiang Uygur autonomous region, Qinghai, Inner Mongolia, Heilongjiang, Jilin, and eastern Liaoning, among other regions. The low‐suitability habitat of *H*. *macrophylla* covered 9.35% of the total area of China, totaling ca. 900,656/km^2^. The region is composed of two climatic intersections, and some areas are affected by a tropical monsoon climate. One is the intersection of temperate continental climate and temperate monsoon climate, the other is the intersection of plateau and subtropical monsoon climate. Central Yunnan and central Taiwan are vulnerable to the subtropical monsoon climate. Medium‐suitability habitat accounted for 11.65% of the national area, a total of ca. 1,122,589/km^2^. High‐suitability habitat accounted for 15.47% of the total area of China, totaling ca. 1,490,135/km^2^, mainly distributed in the east of Yunnan‐Guizhou Plateau and Sichuan Basin, North China Plain, the middle and lower reaches of the Yangtze River Plain, and the south of Nanling‐Wuyi Mountain. Therefore, the suitable habitat distribution of *H*. *macrophylla* is mainly distributed in the subtropical seasonal wind active area, and a small amount is distributed in the temperate and tropical monsoon areas. The leaves of *H*. *macrophylla* are generally prone to wilting in summer when there are high temperatures and limited water.

### 3.3 Future changes in suitable habitat area

**FIGURE 3:**
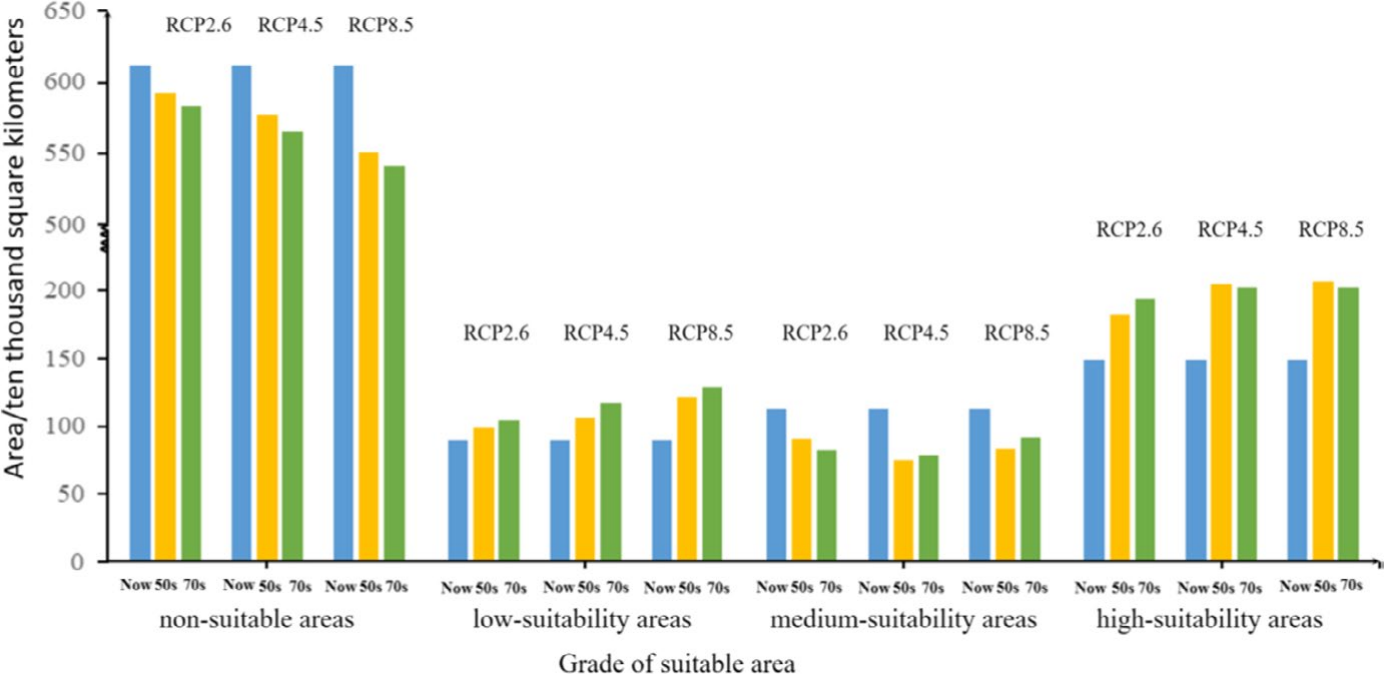
Changes of suitable habitat area of *Hydrangea macrophylla* under three different climate scenarios (RCP2.6, RCP4.5, and RCP8.5) in current times, the 2050s, and 2070s. Classification of suitable areas (non‐suitable areas, low‐suitable areas, medium‐suitability areas, and high‐suitability areas)

**FIGURE 4:**
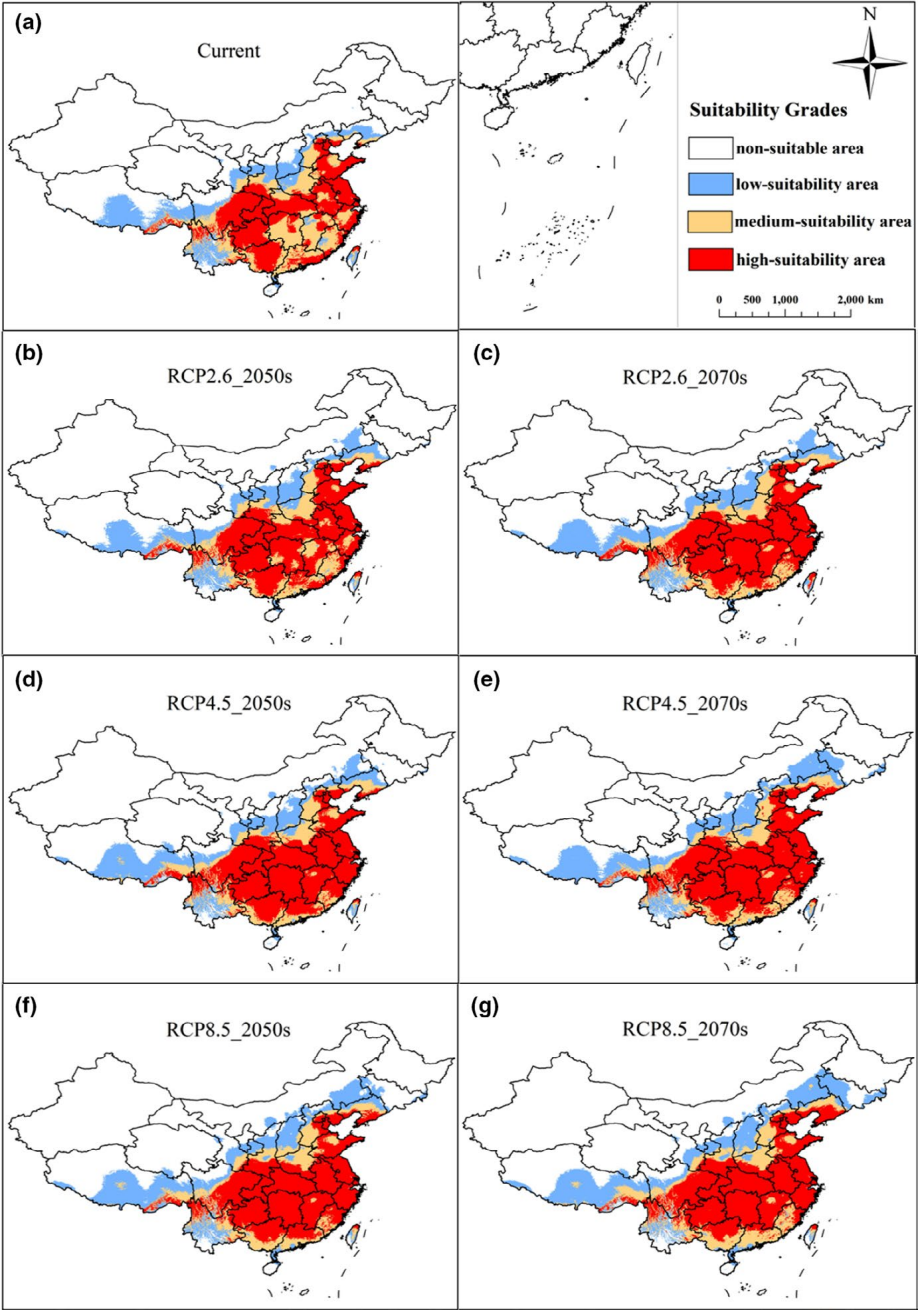
The current and future potential geographical distribution of *Hydrangea macrophylla* by 2050s and 2070s according to the climate scenarios RCP 2.6, RCP4.5, and RCP 8.5 based on optimized parameters. (a) Current; (b) RCP2.6‐2050s; (c) RCP2.6‐2070s; (d) RCP4.5‐2050s; (e) RCP4.5‐2070s; (f) RCP8.5‐2050s; (g) RCP8.5‐2070s

Under different future climate scenarios, four grades of the potential geographical distribution areas vary significantly, but the change in the total suitable area is less than that of current times. As shown in Figures 3 and 4, in the 2050s, under three different emission scenarios (RCP2.6, RCP4.5, and RCP8.5), the area of unsuitable habitat will reduce by 2%, 3.67%, and 6.33%, respectively, compared with that of current times. In the 2070s, the model predicts that the area of unsuitable habitat of *H*. *macrophylla* will be 3.04% (RCP2.6), 4.86% (RCP4.5), and 7.41% (RCP8.5), less than it is currently. This indicates that many unsuitable areas will be replaced by low‐, medium‐, or high‐suitability areas. Therefore, the total suitable area of *H*. *macrophylla* will increase in the future. The area of low‐suitability habitat increases by 0.87% (RCP2.6), 1.70% (RCP4.5), and 3.32% (RCP8.5) in the 2050s. This further increases by 0.58% (RCP2.6), 1.11% (RCP4.6), and 0.72% (RCP8.5) in the 2070s. From current times to the 2070s, the low‐suitability habitat of *H*. *macrophylla* gradually increases, and the range increases gradually with rising greenhouse gas concentration. Under the three different emission scenarios, the area of medium‐suitability habitat decreases by 2.27%, 3.83%, and 3.02%, respectively, from current times to the 2050s. From current times to 2070s, it decreases by 3.11%, 3.46%, 2.15%, respectively. Some of these suitable areas will be transformed into low‐suitability habitat and some into high‐suitability habitat. The high‐suitability habitat area of *H*. *macrophylla* also shows a trend of expansion in the future. By the 2050s, the high‐suitability habitat area increases by 3.40%, 5.79%, and 6.03%, respectively. Compared with the current area, the area of these regions will increase by 4.70%, 5.50%, and 5.51%, respectively, in the 2070s. In general, the change of suitable habitat of *H*. *macrophylla* in the future shows a trend of decreasing non‐suitable habitat and increasing suitable habitat increasing under three different greenhouse gas emission scenarios. At the same time, low‐ and high‐suitability habitat will increase, and medium‐suitability habitat will begin to differentiate.

### 3.4 Analysis of spatial pattern changes

**FIGURE 5:**
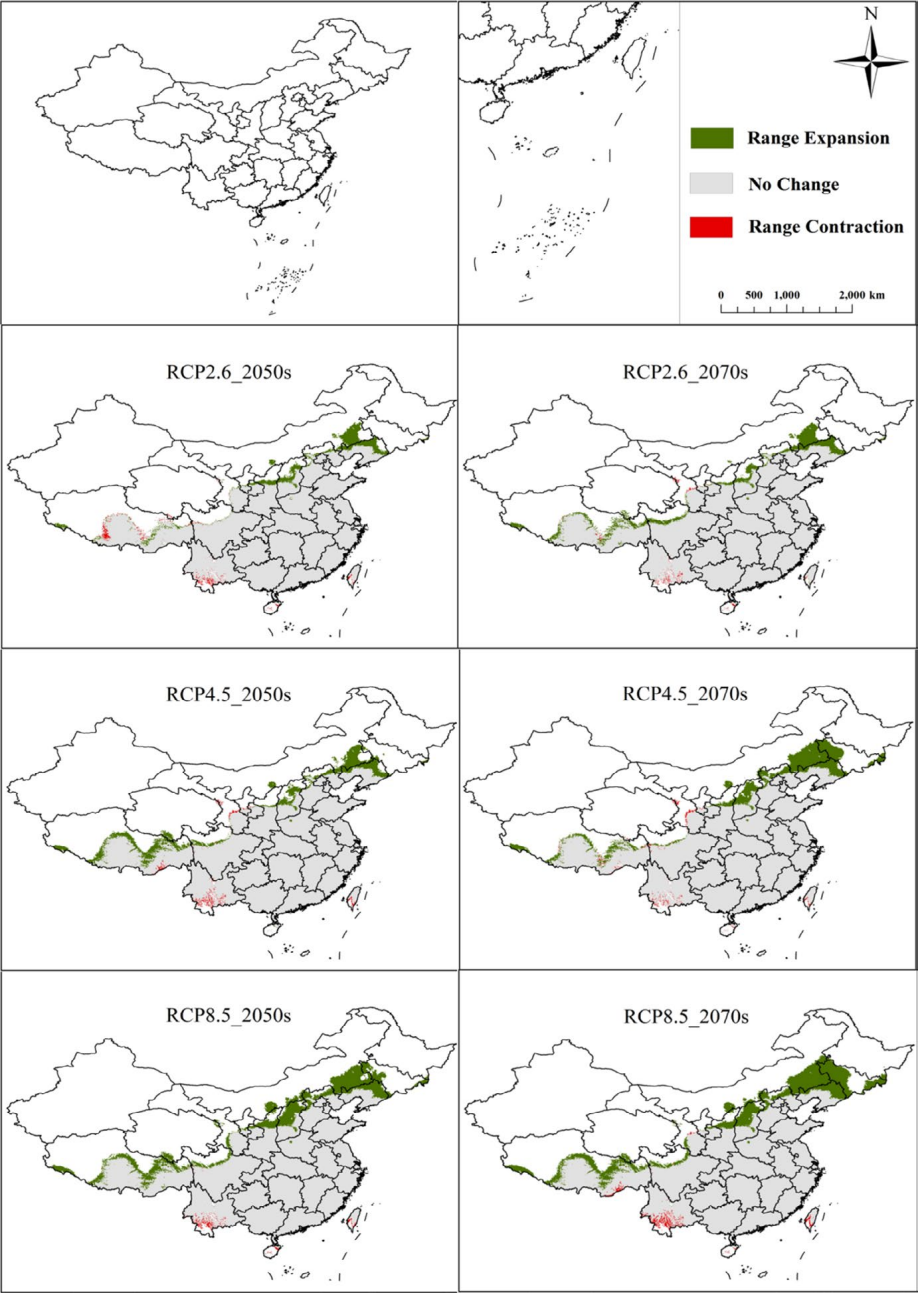
Comparison of changes in the spatial pattern of total suitable *Hydrangea macrophylla* habitat under three different climate scenarios (RCP2.6, RCP4.5, and RCP8.5) in the 2050s and 2070s. Total suitable area change: spruce green represents expansion area, red represents contraction area, gray represents unchanged area, and white represents non‐suitable area

We used the activity analysis method in the SDMToolbox package to track the centroid trajectory of the distribution area of *H*. *macrophylla* with time under different greenhouse gas emission scenarios in order to explain the future displacement and direction change of its distribution (Brown, 2014). Regardless of the greenhouse gas emission scenario, from current times to the future (2070s), the centroid of total suitable habitat area of *H*. *macrophylla* migrates from the southwest of Hubei Province (109°6′41.9004″N, 30°4′2.6112″E) to the north, and finally reaches Chongqing (Figure 7). The total area of suitable habitat expands to the north, while that of the southern region decreases (Figure 5). Under RCP2.6, RCP4.5, and RCP8.5, in the 2050s, it moves northeast by distances of 81.26 km, 82.51 km, and 138.46 km, respectively. And from the current period to 2070s, the centroid moves to the northeast by distance of 73.09 km, 152.58 km, and 186.15 km. The distance increases with the increase of CO_2_ concentration (Appendices 2 and 3).

**FIGURE 6:**
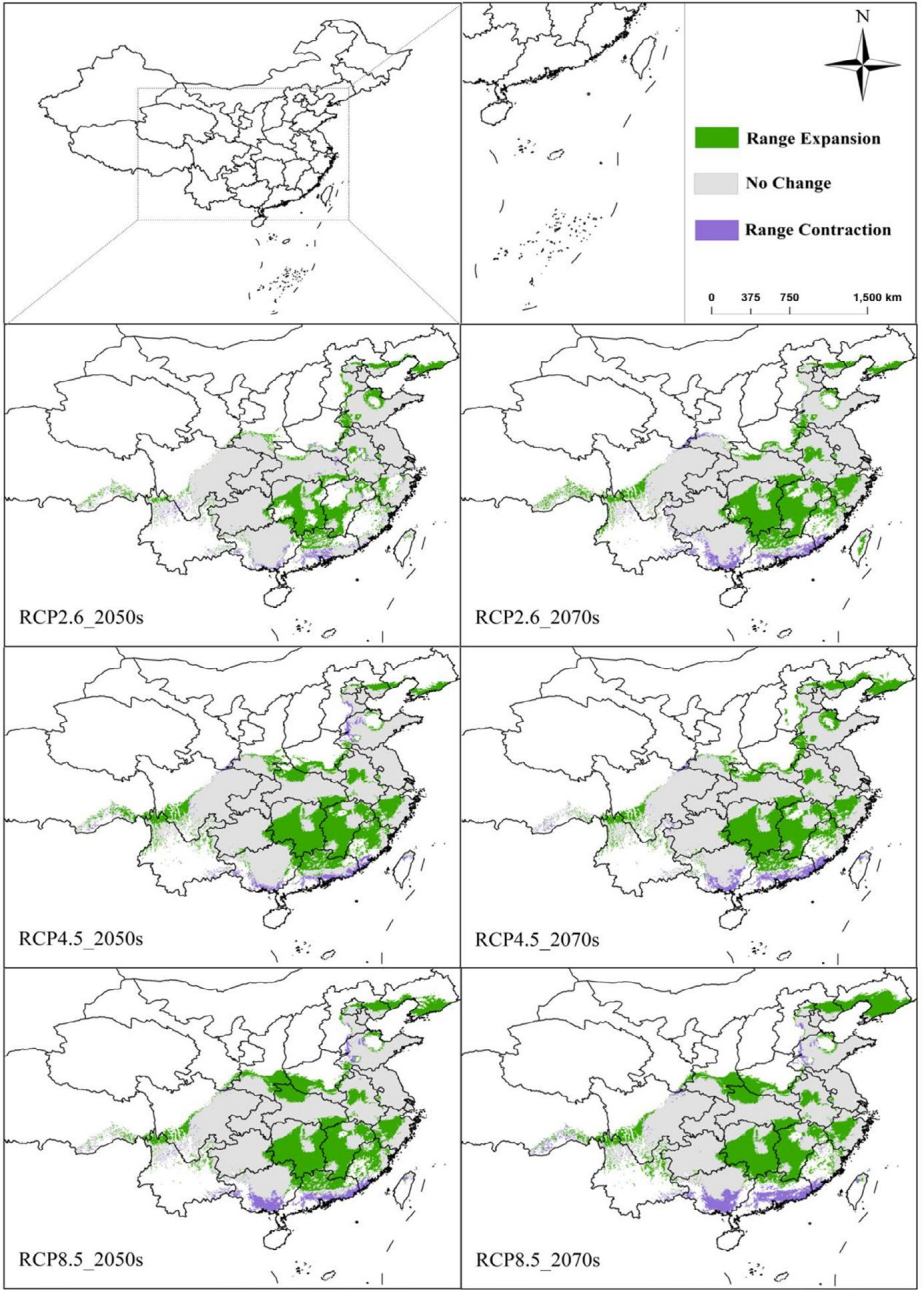
Spatial pattern of the *Hydrangea macrophylla* high‐suitability habitat area in the 2050s and 2070s under three different climate scenarios (RCP2.6, RCP4.5, and RCP8.5). High‐suitability area change: grass green represents expansion area, purple represents contraction area, gray represents unchanged area, and white represents non‐suitable area

**FIGURE 7:**
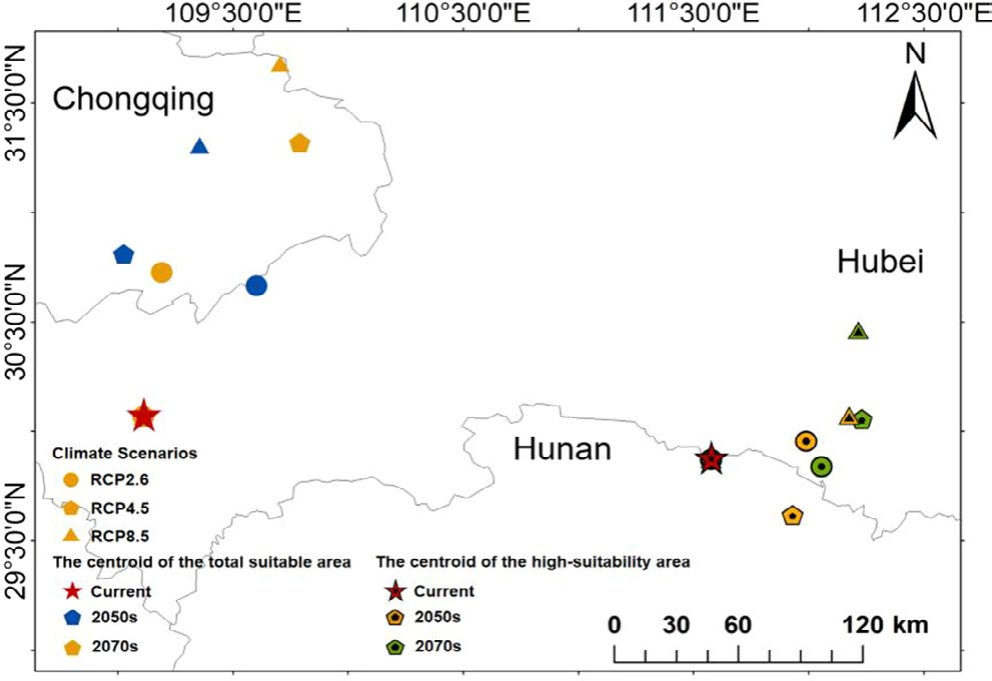
Shift distance of the centroid of *Hydrangea macrophylla* under three different climate scenarios (RCP2.6, RCP4.5, and RCP8.5) in the future (The centroid of total suitable habitat and the centroid of high‐suitability habitat were distinguished by the center of symbols). Climate scenarios were distinguished by a given symbol; for time periods, the current time was displayed by a red star, and the 2050s and 2070s were distinguished by a given color

In general, under the various emission scenarios, the trajectory distance and direction of the centroid change over time. The centroid of total suitable area appears to be located northeast of the present position by the 2070s, while the centroid in the high‐suitability habitat gradually migrates from Hunan Province to Hubei Province. Finally, they will all be located to the east of the current centroid (Figure 7). Combined with Figure 6, the high‐suitability area will expand to the north. In addition, the high‐suitability areas of Hunan, Jiangxi, and Tibet will also increase, while the areas of Guangdong and Guangxi in the South will decrease sharply. At present, the centroid of the high‐suitability area is located in Li County, Changde City, Hunan Province (111°34′34.338″N, 29°52′14.1996″E). Under RCP2.6, it moves to the northeast by the 2050s, with a migration distance of 41.10 km; however, the centroid in the 2070s is in the southeast of the current centroid, with a distance of 46.59 km. Under RCP4.5, the centroid moves southeast from the current position in the 2050s, with a migration distance of 44.33 km; however, the center centroid in the 2070s is located in the northeast of the current centroid, with a distance of 66.51 km. Under RCP8.5, it moves northeast with a migration distance of 62.05 km in the 2050s, and the centroid in 2070s is located in the northeast of the current centroid, with a distance of 89.84 km (Appendix 3). These results showed that the change in the movement of the area of total *H*. *macrophylla* suitable habitat under different greenhouse gas emission scenarios is generally consistent with the movement of its centroid (Figure 7 and Appendix 3). Generally, the centroid movement is consistent with changes in the spatial pattern of the suitable area predicted by the MaxEnt model (Li et al., 2021).

### 3.5 Environmental variables analysis

The results of jackknife test showed that the top three climate variables with the largest influence on the normalized training gain, test gain, and AUC value were as follows: precipitation of the warmest quarter (*Bio18*), min temperature of the coldest month (*Bio6*), and annual precipitation (*Bio12*), when modelling with a single environmental factor (Appendix 4). Furthermore, the contribution rate of environmental variables can also be used to evaluate the importance of the environment. The contribution rate of each environmental variable to the distribution of *H*. *macrophylla* was combined (Table 3), the top three bioclimatic variables with their contribution rates were min temperature of the coldest month (*Bio6*, 33.64%), precipitation of the warmest quarter (*Bio18*, 23.53%), and temperature seasonality (*Bio4*, 22.32%), and the cumulative value of the three items reached 79.49%. Therefore, precipitation of the warmest quarter (*Bio18*), min temperature of the coldest month (*Bio6*), and temperature seasonality (*Bio4*), annual precipitation (*Bio12*), were the main environmental variables affecting *H*. *macrophylla* habitat suitability.

**TABLE 3:** Contribution of the environmental predictors influencing the probability of distribution of *Hydrangea macrophylla* in China

Climate variables	Percentage contribution (%)	Climate variables	Percentage contribution (%)
Min temperature of coldest month	33.6443	Precipitation of driest quarter	2.3139
Precipitation of warmest quarter	23.5274	Isothermality	2.0843
Temperature seasonality	22.3204	Precipitation seasonality	1.7329
Mean diurnal range	8.8186	Mean temperature of wettest quarter	1.287
Temperature annual range	2.9875	Annual precipitation	1.2873

It is generally believed that when the existence probability exceeds 0.5, the corresponding ecological factors are suitable for plant growth (Guo et al., 2017). When precipitation of the warmest quarter (*Bio18*) reached 816 mm, the distribution probability peaked. Subsequently, with the increase of precipitation of the warmest quarter (*Bio18*), the probability of *H*. *macrophylla*'s existence began to decline. If an existence probability >0.5 is taken as the suitable range (Jia et al., 2019), the suitable range of the warmest quarter was 324–1272 mm. Similar to the precipitation of the warmest quarter (*Bio18*), the existence probability of *H*. *macrophylla* increased with the increase of the min temperature of the coldest month (*Bio6*), and annual precipitation (*Bio12*), while they would decrease after reaching the peak. With the probability of existence greater than 0.5 as the suitable range, the suitable range of *Bio6* was about −13.1~4.2°C, and the suitable range of *Bio12* was about 872.7~4090.6 mm.

## 4 DISCUSSION

### 4.1 Predictive capabilities of the MaxEnt model

The MaxEnt model has been widely used in ecology, conservation biology, evolutionary biology, and invasive species management (Phillips et al., 2006). It has many advantages, such as wide application range, high precision, simple operation, low sample number requirement, and stable operation results (Li et al., 2020).

*Hydrangea macrophylla* is the most popular flowering shrub that is widely used in landscape gardening and has crucial commercial value (Lisa, 2019). As a globally introduced plant species, a less studied but important characteristic is its adaptability to the environment. The key environmental factors affecting or limiting its growth should be studied by assessing its species distribution patterns. Based on the results of the jackknife test and the contribution rate of climate variables calculated by the model, we determined the main environmental factors affecting the variation in *H*. *macrophylla* distribution. The predicted results showed that the variables involved in the regional changes of suitable habitat for *H*. *macrophylla* were as follows: min temperature of the coldest month (*Bio6*), precipitation of the warmest quarter (*Bio18*), annual precipitation (*Bio12*), and temperature seasonality (*Bio4*). Therefore, its distribution was determined by both temperature and precipitation factors. Both high temperature and low temperature stress could affect its growth. When *H*. *macrophylla* was stressed at high temperature, a series of changes occurred in its internal physiological mechanism, which mainly showed chlorophyll degradation, increased relative permeability of cell membrane, and increased MDA content (Xin & Shi, 2009). When the environmental temperature continued to be lower than the minimum temperature of plant growth, it would cause freezing damage to cold‐resistant plants, which not only limits the geographical distribution of plants, but also seriously affects the yield and ornamental value of plants (Pagter et al., 2008). *H*. *macrophylla* is a very popular flowering plant, low temperature or frozen soil will seriously damage the buds, thus considerably reducing the attractiveness of *H*. *macrophylla* to consumers (Ren et al., 2020). In addition, precipitation is one of the most important environmental factors affecting plant growth, and it is an important factor in the survival and growth of seedlings (Liu, 2020). Abundant rainfall leads to a significant increase in soil water content near the planting site, providing an excellent growth environment for *H*. *macrophylla*, which has a large water demand. However, if the rainfall is too much, the waterlogging stress will lead to the closure of stomata, the decrease of photosynthetic capacity and the increase of respiratory energy consumption, which is not conducive to the accumulation of organic matter. But some varieties of *H*. *macrophylla* had strong adaptability to flooding stress, and could be planted in waterfront, low‐lying wetland, and other sites (Zhang et al., 2019). In the arid environment, the leaf transpiration of *H*. *macrophylla* was high, and even a short period of wilting would lead to the visual phenomena of leaf edge drying and flower necrosis, which would lead to the decrease of yield, quality, or ornamental value (Ren et al., 2020). The harm degree of environmental factors to *H*. *macrophylla* was also related to the length of time it was under stress. When *H*. *macrophylla* is under high temperature or drought stress in the early‐life stages, it can resist stress damage by adjusting its osmotic regulation substances and protective enzyme activities; however, when the stress duration increases by a certain extent, the damage caused is irreversible. The damage of double stress is far greater than that of single stress (Sun et al., 2019). All of these stress phenomena corresponded to the main limiting factors screened out in this paper.

Therefore, in the high temperature and rainy areas of southern China, the distribution pattern of *H*. *macrophylla* would shift frequently in the future. The existing research results could provide a reliable theoretical basis for the prediction of MaxEnt model. However, the prediction of this model is faster and more systematic than traditional experimental methods (Ashraf et al., 2016).

### 4.2 Changes in spatial pattern and centroid of potential *Hydrangea macrophylla* distribution

The overall change in suitable habitat area of *H*. *macrophylla* was as follows: from the current time to 2070s, the total suitable habitat area would continue to expand with the increase of greenhouse gas emission. It was consistent with the results of a study on the Chinese Sea Buckthorn (*Hippophae rhamnoides* subsp. *sinensis*) suitable area, which the planting potential of this shrub in the high emission scenario was greater than that in the low emission scenario (Huang et al., 2018). A study on the distribution of the alpine vegetation distribution in the Greater Himalaya also showed that under the high concentration emission scenario RCP 8.5, the low‐ and high‐suitability habitat for shrub species would be increased in the middle of the 21st century with the increase of CO_2_ concentration (Bashir, 2021). A similar expansion was also expected in the western Himalayas under future global warming (Changjun et al., 2021). The reason why the distribution area would expand in the future is that the climate in China would transition to warm and humid under the influence of the greenhouse effect induced by the increase of CO_2_ concentration. However, the change of the medium‐suitability areas of *H*. *macrophylla* was not completely consistent with the change of the above area. Regardless of types of greenhouse gas emission scenario, the medium‐suitability area of *H*. *macrophylla* in the future would reduce compared with the current area, similar to the medium‐suitability habitat of [*Actinidia chinensis* Planch.] in China (Zhang et al., 2017).

Under climate change scenarios, changes in the centroid of a plant's distribution area would reflect the distance and direction of the distribution area movement. Centroid analysis based on the SDMToolbox plug‐in showed that the centroid of the total suitable habitat of *H*. *macrophylla* was currently located in Lichuan City, Enshi Tujia and Miao Autonomous Prefecture, Hubei Province (109°6′41.9004″N, 30°4′2.6112″E). And the centroid of the high‐suitability habitat was located in Changde City, Hunan Province (111°34′34.338″N, 29°52′14.1996″E). The centroid of the total suitable area of *H*. *macrophylla* showed a trend of migration to the north under three different greenhouse gas emission scenarios (2050s and 2070s), which was consistent with the conclusion of previous studies that climate warming would result in plant migration to high‐altitude and high‐latitude areas (Ashraf et al., 2016; Wardle & Coleman, 2011; Wu et al., 2021). Only under RCP2.6‐2070s and RCP4.5‐2050s, the centroid of the highly suitability habitat moved to the southeast. But under the scenarios of other periods, it was consistent with that of the total suitable area and moved to the north. Combined the trajectory distance and direction of centroid in different periods, we speculated that the centroid would not always move in the same direction, and the smaller the time span, the more complex its trajectory is, which was similar to a previous study on other plant (Wang, Li, et al., 2018; Wang, Wu, et al., 2018). In addition, from the perspective of model prediction, the centroid of *H*. *macrophylla* distribution areas moved in different directions over time, which indicated that future climate change would not only result in the distribution areas shifting northward, but also led to significant changes in the distribution pattern of *H*. *macrophylla*. The environmental requirements of plants living in the central area of *H*. *macrophylla* distribution might be different from those living in the marginal areas. In order to better understand the future distribution of *H*. *macrophylla*, we need to further study the ecological and physiological mechanisms of its adaptation to environmental factors.

### 4.3 Study limitations

The first limitation of this study was that the 10 environmental variables selected cannot reflect all the factors affecting the geographical distribution of *H*. *macrophylla*. In addition to the selected factors, other abiotic factors, such as light, soil, and air; biological factors, such as the interaction between species; and anthropogenic impacts all have a significant impact on the species distribution (Qin et al., 2020). Secondly, most species distribution models speculate the environmental conditions of species based on the observed species occurrence data. Therefore, we cannot simply equate the habitat of species occurrence data with the suitable habitat for a species, that is, the suitable habitat area predicted by the model does not necessarily contain the species (Li et al., 2013). Uncertainties such as the internal assumptions of species distribution models need to be considered and further studied.

## 5 CONCLUSIONS

Although the MaxEnt model still requires verification and revision by further biological research, the basic conclusion of this study is generally consistent with most of the current research, that is, with the enhancement of the global greenhouse effect, most plants growing in the northern hemisphere will move to high latitudes in the future. Therefore, the relevant companies (such as those developing Huahai tourism) and institutions (such as individuals and units collecting *H*. *macrophylla* germplasm resources) should consider these predicted changes. For the new suitable areas of *H*. *macrophylla* in the future, the relevant departments should formulate reasonable and sustainable land use planning in combination with the local environment, in order to leave sufficient space for its eventual migration. As the new suitable areas are scattered on the edge of the suitable areas, in order to increase the possibility of *H*. *macrophylla* migration, human interference activities should be reduced as far as possible in these areas. For the lost suitable areas of *H*. *macrophylla*, active *ex situ* protection measures should be taken, and botanical gardens should be established to transplant *Hydrangea* in artificial environment for cultivation, maintenance, and preservation. According to the environmental factors restricting the distribution of *H*. *macrophylla*, an artificial environment suitable for its growth should be built, and even artificial afforestation should be carried out to maximize the ornamental, medicinal, and economic values of *Hydrangea*. The preservation of suitable habitat areas can provide a refuge for *H*. *macrophylla* to adapt to climate change in the future.

Based on the prediction of the MaxEnt model, our research indicated that *H*. *macrophylla* in China might be affected by future climate change and its total suitable habitat area would gradually expand. Among them, medium‐suitability habitat would gradually differentiate and reduce, and the area of low‐ and high‐suitability habitat would gradually expand. Under three different emission scenarios, the suitable habitat area of *H*. *macrophylla* would all expand to the north. These findings are crucial for the development of climate change adaptation strategies for this plant. Further studies on its biological and ecological adaptability should be carried out according to the different suitable habitat areas in the future and the plant's response to climate change.

## CONFLICT OF INTEREST

The authors declare that they have no conflict of interest.

## AUTHOR CONTRIBUTIONS

**Xingyue Yan:** Conceptualization (equal); Data curation (lead); Formal analysis (lead); Investigation (lead); Methodology (lead); Project administration (equal); Resources (lead); Software (lead); Supervision (equal); Validation (lead); Visualization (lead); Writing‐original draft (lead); Writing‐review & editing (lead). **Shuchen Wang:** Conceptualization (supporting); Investigation (supporting); Software (supporting). **Yu Duan:** Software (supporting); Visualization (supporting). **Jing Han:** Data curation (supporting); Methodology (supporting). **Donghua Huang:** Methodology (supporting); Visualization (supporting). **Jian Zhou:** Formal analysis (supporting); Funding acquisition (lead); Project administration (supporting); Supervision (lead); Validation (supporting).

## Data Availability

All date in this manuscript are available on Dryad (https://doi.org/10.5061/dryad.6hdr7sr1z).

## APPENDIX 1

Parameters of the candidate models

**Table:**

Regularization multipliers	0.5, 1, 1.5, 2, 2.5, 3, 3.5, 4
Feature classes	L, Q, P, T, H, LQ, LP, LT, LH, QP, QT, QH, PT, PH, TH, LQP, LQT, LQH, LPT, LPH, QPT, QPH, QTH, PTH, LQPT, LQPH, LQTH, LPTH, LQPTH

## APPENDIX 2

Centroid coordinates for total and highly suitable areas of *Hydrangea macrophylla* under three different climate scenarios

**Table:**

	1970–2000	RCP2.6		RCP4.5		RCP8.5	
		2050s	2070s	2050s	2070s	2050s	2070s
The total suitable areas							
Longitude	109°6′41″N	109°36′8″N	109°11′32″N	109°1′38″N	109°47′29″N	109°21′19″N	109°42′23″N
Latitude	30°4′2″E	30°39′55″E	30°43′31″E	30°48′40″E	31°19′7″E	31°18′24″E	31°40′44″E
The highly suitable areas							
Longitude	111°34′34″N	111°59′27″N	112°3′27″N	111°55′30″N	112°13′53″N	112°10′35″N	112°12′56″N
Latitude	29°52′14″E	29°57′18″E	29°50′22″E	29°37′3″E	30°3′23″E	30°4′17″E	30°27′37″E

## APPENDIX 3

Distance and direction of centroid movement for total and highly suitable areas of *Hydrangea macrophylla* under three different climate scenarios (RCP2.6, RCP4.5, and RCP8.5)

**Table:**

	The total suitable areas				The highly suitable areas			
	Current to 2050s		Current to 2070s		Current to 2050s		Current to 2070s	
	Distance (km)	Direction	Distance (km)	Direction	Distance (km)	Direction	Distance (km)	Direction
RCP2.6	81.26	Northeast	73.08	Northeast	41.10	Northeast	46.59	Southeast
RCP4.5	82.51	Northwest	152.58	Northeast	44.33	Southeast	66.51	Northeast
RCP8.5	138.46	Northeast	186.15	Northeast	62.05	Northeast	89.84	Northeast

## APPENDIX 4

Relative predictive power of different environmental variables based on the jackknife of AUC for *Hydrangea macrophylla*. The gain (%) with all predictors = Red bar; the gain (%) with only predictor = Blue bar; the gain (%) without the corresponding predictor = Green bar
